# Lactate Provides Metabolic Substrate Support and Attenuates Ischemic Brain Injury in Mice, Revealed by ^1^H-^13^C Nuclear Magnetic Resonance Metabolic Technique

**DOI:** 10.3390/biomedicines13040789

**Published:** 2025-03-24

**Authors:** Kefan Wu, Yajing Liu, Yuxuan Wang, Jiabao Hou, Meng Jiang, Shaoqin Lei, Bo Zhao, Zhongyuan Xia

**Affiliations:** Department of Anesthesiology, Renmin Hospital of Wuhan University, Wuhan 430064, China; wkf7788@whu.edu.cn (K.W.); liuyj0204@163.com (Y.L.);

**Keywords:** NMR, cerebral ischemia, lactate, glycolytic

## Abstract

**Background/Objectives:** Lactate, classically considered a metabolic byproduct of anaerobic glycolysis, is implicated in ischemic acidosis and neuronal injury. The recent evidence highlights its potential role in sustaining metabolic networks and neuroprotection. This study investigates lactate’s compensatory mechanisms in ischemic brain injury by analyzing post-ischemic metabolic enrichments and inter-regional metabolite correlations. **Methods:** Dynamic metabolic profiling was conducted using ^13^C-labeled glucose combined with ^1^H-^13^C NMR spectroscopy to quantify the metabolite enrichment changes in a murine cerebral ischemia model (*n* = 8). In vivo validation included intracerebroventricular pH-neutral lactate infusion in ischemic mice to assess the behavioral, electrophysiological, and mitochondrial outcomes. In vitro, HT22 hippocampal neurons underwent oxygen–glucose deprivation (OGD) with pH-controlled lactate supplementation (1 mM), followed by the evaluation of neuronal survival, mitochondrial membrane potential, and glycolytic enzyme expression. **Results:** NMR spectroscopy revealed a 30–50% reduction in most cerebral metabolites post-ischemia (*p* < 0.05), while the quantities of lactate and the related three-carbon intermediates remained stable or increased. Correlation analyses demonstrated significantly diminished inter-metabolite coordination post-ischemia, yet lactate and glutamate maintained high metabolic activity levels (r > 0.80, *p* < 0.01). Lactate exhibited superior cross-regional metabolic mobility compared to those of the other three-carbon intermediates. In vivo, lactate infusion improved the behavioral/electrophysiological outcomes and reduced mitochondrial damage. In the OGD-treated neurons, pH-neutral lactate (7.4) reduced mortality (*p* < 0.05), preserved the mitochondrial membrane potential (*p* < 0.05), and downregulated the glycolytic enzymes (HK, PFK, and PKM; *p* < 0.01), thereby attenuating H^+^ production. **Conclusions:** Under ischemic metabolic crisis, lactate and the three-carbon intermediates stabilize as critical substrates, compensating for global metabolite depletion. pH-neutral lactate restores energy flux, modulates the glycolytic pathways, and provides neuroprotection by mitigating acidotoxicity.

## 1. Introduction

Cerebral ischemic injury remains the leading cause of mortality and long-term disability worldwide, particularly in perioperative settings where hemodynamic instability or thromboembolic events frequently precipitate brain hypoperfusion [1]. The current therapeutic strategies, such as thrombolysis and thrombectomy, are constrained by narrow time windows (<4.5–6 h post-onset), leaving many patients with irreversible neuronal damage [2,3]. The brain’s exquisite sensitivity to blood flow disruption—neuronal energy failure occurs within minutes of ischemia—exacerbates the outcomes, highlighting the urgent need to identify the endogenous neuroprotective mechanisms that operate beyond acute reperfusion [4].

Historically, lactate accumulation has been viewed as a biomarker of anaerobic glycolysis and a contributor to ischemic acidosis, correlating with poor prognosis. However, the emerging evidence challenges this paradigm, suggesting lactate may play adaptive roles in metabolic stress. For instance, exercise-induced lactate production enhances hippocampal neurogenesis and memory consolidation via monocarboxylate transporter (MCT)-dependent mechanisms [5]. Similarly, lactate stabilizes HIF-1α to promote angiogenesis in ischemic tissues [6], while acidic microenvironments—rather than lactate itself—drive neuronal injury through proton-sensitive ion channels [7,8]. These findings imply that lactate’s effects are context-dependent, with pH neutrality being critical for its beneficial actions.

Central to this duality is the lactate shuttle hypothesis, which posits lactate as a dynamic energy substrate coordinating intercellular and inter-organ metabolic communication. In the brain, astrocytes export lactate to fuel neuronal oxidative metabolism under physiological conditions, a process termed the astrocyte–neuron lactate shuttle (ANLS) [9]. During ischemia, this shuttle may adapt to sustain energy flux, though its role remains contentious. While some studies implicate lactate accumulation in excitotoxicity [10,11,12], others demonstrate lactate infusion improves the outcomes in experimental stroke [13]. Resolving this controversy requires precise metabolic mapping to disentangle lactate’s pH-dependent effects from global acid–base disturbances.

To address this gap, we employed ^13^C–glucose tracing (Longteng Biotechnology Co., Ltd., Qingdao, China) coupled with ^1^H-^13^C NMR spectroscopy, a technique offering unparalleled resolution for tracking real-time metabolic fluxes and spatial metabolite distribution without radiation exposure [14,15,16]. Unlike positron emission tomography (PET) or mass spectrometry, NMR enables the simultaneous quantification of multiple metabolites and isotopic enrichment, critical for analyzing cross-regional metabolic coordination [17,18]. By integrating in vivo metabolic profiling with in vitro models of pH-controlled lactate supplementation, we elucidated lactate’s neuroprotective role in ischemic injury and identified lactate as a critical metabolic substrate under cerebral ischemic conditions.

## 2. Materials and Methods

### 2.1. Animals

This study utilized specific pathogen-free (SPF) C57BL/6J male mice aged 8–12 weeks (body weight: 25–32 g) provided by Changsheng Biotechnology Co., Ltd. (Liaoning, China). The mice were housed under a 12 h light/dark cycle at 22 °C ± 2 °C and 40–60% humidity, with free access to food and water. Three mice per cage were housed on sterile woodchip bedding, which was replaced every 36 h. All the animals underwent regular health checks, and those showing lethargy or abnormalities were excluded to ensure data reliability. The mice were randomly assigned to three groups (*n* = 8/group). The control group received [3-^13^C] glucose (200 mg/kg) via tail vein injection. The ischemia group underwent [3-^13^C] glucose injection (200 mg/kg) for 30 min, followed by common carotid artery occlusion (CCAO) to induce acute cerebral ischemia.

All mice fasted for 12 h prior to surgery to standardize the metabolic baseline conditions. Anesthesia was administered and maintained with sevoflurane (4% induction; 2% maintenance) via tracheal intubation, supplemented with a rodent ventilator to ensure stable respiration. Following hair removal and the povidone–iodine disinfection of the cervical region, a midline incision was made, and blunt dissection was performed to expose the common carotid arteries (CCAs) using microforceps. In the ischemia group, the bilateral common carotid arteries were occluded with non-traumatic microvascular clamps (0.3 mm tip) for 30 min to induce acute hemispheric ischemia, followed by clamp removal to allow for reperfusion. The incision was sutured, and the mice were placed on a warming pad for recovery. The sham-operated controls underwent the identical procedure without arterial occlusion. Core body temperature was maintained at 37.0 ± 0.5 °C using a heating pad, and blood glucose levels were monitored pre-occlusion and pre-euthanasia, with mice exhibiting fluctuations <20% excluded from analysis.

### 2.2. NMR

#### 2.2.1. Sample Preparation and Tissue Extraction

The mice were euthanized post-surgery via microwave fixation (high power, 13 s; Galanz, Foshan, China) to rapidly terminate brain metabolism. Brain tissues were promptly dissected into functional regions: the frontal cortex (FC), the occipital cortex (OC), the parietal cortex (TC), the striatum (STR), the hippocampus (HP), and the thalamus (THA). The left liver lobe fragment was collected for supplementary metabolic validation. All tissues were immediately weighed and flash-frozen at −80 °C [19].

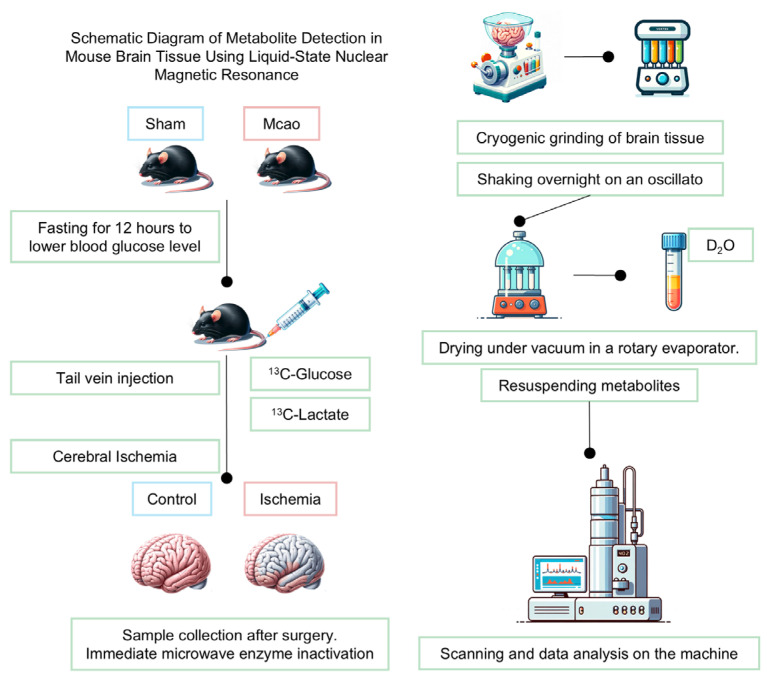



For metabolite extraction, a methanol/ethanol dual-phase protocol was employed. Each sample was homogenized at 20 Hz for 90 s in 200 μL ice-cold 0.1 M HCl/methanol, followed by the addition of 800 μL 60% ethanol and repeat homogenization. After centrifugation (14,000× *g*, 10 min, 4 °C), the supernatants were pooled. The extraction was repeated twice with 1200 μL 60% ethanol. The combined supernatants were vacuum-concentrated (methanol/ethanol removal) and lyophilized.

The lyophilized residues were reconstituted in 600 μL D2O-based NMR buffer (0.2 M Na_2_HPO_4_/NaH_2_PO_4_, pH 7.2) containing 5 mM TMSP (3-(trimethylsilyl)-propionate-2,2,3,3-d4 sodium salt) as an internal standard. The samples were vortexed, centrifuged (14,000× *g*, 15 min), and 500 μL supernatants was transferred to NMR tubes for analysis.

#### 2.2.2. NMR Spectrum Acquisition and Data Processing

All NMR spectra were acquired at 298 K in random order using a Bruker Avance III 500 MHz spectrometer equipped (Karlsruhe, Germany) with a 5 mm TCI cryoprobe. The [^1^H-^13^C]-NMR experiments employed a POCE (Proton-Observed Carbon-Edited) pulse sequence optimized for detecting ^13^C-labeled metabolites. Two complementary scans were performed. A reference scan (without carbon inversion pulse) detected total metabolite signals (^12^C + ^13^C). An edited scan (with carbon inversion pulse) isolated the ^13^C-labeled metabolites by subtracting ^12^C signals. The key acquisition parameters included echo time: 8 ms; spectral width: 20 ppm; relaxation delay: 20 s; scans: 64; and data points: 64 k.

Raw spectra underwent phase correction and baseline correction using Topspin 2.1 (Bruker, Karlsruhe, Germany). Subsequent processing utilized the R package NMRSpec for the automated integration of ^13^C-enriched peak regions (Table 1) and isotopic enrichment quantification [20]. Specific integration boundaries were defined based on known chemical shifts (±0.05 ppm tolerance) to minimize the cross-talk between adjacent peaks.

In NMR analysis, metabolites were identified and quantified based on characteristic chemical shifts, with numerical suffix (e.g., GABA3 and Glu4) indicating specific carbon position labeled by ^13^C. Key metabolites included TMSP (3-(trimethylsilyl)propionate-2,2,3,3-d4 sodium salt, internal standard), GABA (γ-aminobutyric acid; GABA3 and GABA4 denote C3 and C4 labeling), NAA (N-acetylaspartate), NAAG (N-acetylaspartylglutamate), glutamate (Glu3 and Glu4 for C3/C4 labeling), glutamine (Gln4 and C4-labeled), Glx (glutamate–glutamine pool; Glx2 and Glx3 refer to C2/C3 labeling), Suc (succinate), Asp (Aspartate; Asp3 and C3-labeled), m-Ins (myo-inositol; m-Ins2 and m-Ins3 specify hydroxyl positions), and Tau (taurine). Alanine (Ala) was included as glycolytic intermediate. Labeling positions were verified against ^13^C isotopic enrichment patterns derived from [3-^13^C] glucose.

### 2.3. Preparation and pH Measurement of Lactate Solution

#### 2.3.1. Stereotaxic Lateral Ventricular Cannula Implantation

Mouse implantation surgery was completed three weeks before the experiment, and the mice’s condition was closely observed. To bypass the hepatic first-pass metabolism of lactate (30–60% systemic clearance observed in NMR pilot studies), a dual-lumen cannula with lateral pressure-release ports (Kedou Brain Machine Co., Suzhou, China) was stereotactically implanted into the lateral ventricle. The mice (C57BL/6) under isoflurane anesthesia (2–3%) were fixed on a stereotaxic frame, with coordinates targeting the left lateral ventricle (relative to bregma: anteroposterior [AP] −0.5 mm; mediolateral [ML] +1.0 mm; dorsoventral [DV] −2.0 mm), contralateral to the ischemic hemisphere induced by transient common carotid artery occlusion (tCCAO). The cannula (outer diameter: 1.2 mm; infusion lumen: 0.3 mm; pressure-balanced lumen: 0.2 mm) was secured using three titanium cranial screws (1 mm diameter) interconnected with dental acrylic. The screw terminals extended superficially to interface with an external receiver for continuous field potential monitoring during ischemic episodes. Post-implantation recovery (72 h) preceded neurological assessments.

#### 2.3.2. pH-Buffered Lactate Solution Preparation

Artificial cerebrospinal fluid (aCSF) was formulated to mimic murine CSF ionic composition: NaCl (147 mM), KCl (2.7 mM), CaCl_2_ (1.2 mM), MgCl_2_ (0.85 mM), and NaHCO_3_ (25 mM). Solutions were equilibrated with 5% CO_2_/95% O_2_ (carbogen) to stabilize pH at 7.4. Sodium L-lactate (Abcam, Cambridge, UK, ≥99% purity) was dissolved in pre-equilibrated aCSF (final concentration: 1 mM) to avoid acidosis from free lactic acid. The prepared solutions were sterile-filtered (0.22 μm PVDF membrane), aliquoted, and stored at −20 °C (≤2 freeze–thaw cycles). The mice were randomized into four groups (*n* = 6/group). Sham controls: tCCAO surgery without ischemia induction. Ischemia-only: tCCAO with 20 min occlusion. Ischemia + aCSF: tCCAO with ventricular aCSF infusion. Ischemia + lactate: tCCAO with 1 mM sodium L-lactate in aCSF. During ischemia, the solutions were infused via the cannula’s primary lumen (400 nL/min for 30 min) using a microsyringe pump (Harvard Apparatus, Holliston, MA, USA), while the secondary lumen maintained intracranial pressure equilibrium. After homogenizing the mouse brain tissue, pH was measured, and lactate concentration was detected using a lactate assay kit.

### 2.4. Behavioral Assessments

#### 2.4.1. Open Field Test (OFT)

The open field arena (50 cm × 50 cm × 40 cm, opaque polyethylene) was illuminated uniformly at 50 lux (neutral white LED) in a sound-attenuated room. The mice were acclimated to the testing room for 30 min under dim light (10 lux) prior to the trials. Each mouse was placed in the center of the arena and allowed to explore freely for 10 min. Activity was recorded using a ceiling-mounted camera (Panasonic HC-V380, 30 fps, Osaka, Japan).

#### 2.4.2. Novel Object Recognition (NOR)

The same dimensions as OFT were used, but with distinct visual cues on the walls for spatial orientation. Two identical cubes (3 cm^3^, white acrylic, and sanitized with 70% ethanol) in the training phase; one cube was replaced with a novel pyramid (3 cm height and black ceramic) in the testing phase. The mice freely explored the empty arena for 10 min. Two identical cubes were placed 20 cm apart in the arena. The mice explored for 5 min; exploration was defined as nose contact <2 cm or direct paw touches. The mice with <10 s total object exploration were excluded. One cube was replaced with the novel pyramid. The mice explored freely for 5 min; exploration time for each object was recorded. Discrimination index (DI): (T_novel_ − T_familiar_)/(T_novel_ + T_familiar_). The arena was cleaned with 70% ethanol between trials to eliminate odor cues.

#### 2.4.3. Corner Test

The corner test apparatus consisted of a triangular arena (30 cm × 30 cm × 30 cm) formed by two transparent acrylic walls intersecting at a 30° angle, with a small opening (3 cm × 3 cm) at the vertex to facilitate entry. The mice were gently positioned facing the vertex opening and allowed to explore freely. A trial began when all four paws of each mouse entered the defined corner zone (within 5 cm of the vertex) and ended upon exit. Over 10 consecutive trials, the turning direction (body rotation >90° relative to the vertex axis upon exiting) was recorded, with left/right turn percentages calculated as the ratio of turns to one side relative to total valid trials.

#### 2.4.4. Elevated Beam Test

Motor coordination was assessed using a custom elevated beam (1 m long and 50 cm height) with adjustable widths (12 mm for training and 6 mm for testing) and a darkened escape platform containing food and water at the distal end. The mice were placed at the beam’s starting point, and traversal time (from beam entry to platform arrival) was measured. Trials were terminated at 60 s if the mouse failed to complete the task or fell onto the padded surface below, with such instances recorded as 60 s. Three trials per mouse were conducted under controlled red light conditions to minimize stress.

### 2.5. Neuronal Golgi Staining

We performed Golgi staining on the brain tissue to observe subtle pathological changes in neurons. After completing the experiments, the mice were euthanized, and their brains were perfused to remove excess blood. The brain tissue was fixed in 4% paraformaldehyde dissolved in phosphate-buffered saline (PBS) for 24 h. The tissue was then incubated in Golgi–Cox solution at room temperature for 72 h, with multiple solution changes during the incubation period. Following incubation, the brain tissue was transferred to a 30% sucrose solution in PBS for cryoprotection. The tissue was sectioned into 100 µm thick slices and mounted onto gelatin-coated slides. After staining, the sections were dehydrated, cleared, and covered with a coverslip. Neuronal structures, including dendritic spines and axons, were visualized under a confocal microscope. The morphology of the neurons, including dendritic branching and spine density, was determined using manual counts of protrusions (>0.5 µm length) per 10 µm dendritic segment in tertiary branches (ImageJ v1.53) [21].

### 2.6. Cell Culture

#### 2.6.1. Cell Line Validation

The murine hippocampal neuronal cell line HT22 (Procell Life Science, CL-0605, Hyderabad, India) was authenticated via short tandem repeat (STR) profiling (PowerPlex^®^ 21 System, Promega, Madison, WI, USA) by the supplier and confirmed negative for mycoplasma (MycoAlert™ PLUS, Lonza LT07-710, Basel, Switzerland). The cells were cultured in high-glucose DMEM (Gibco, C11995500BT, Waltham, MA, USA) supplemented with 10% heat-inactivated fetal bovine serum (FBS; Gibco, 10270-106), 100 U/mL penicillin, and 100 µg/mL streptomycin (Gibco, 15140122) at 37 °C under 5% CO_2_ (HERAcell™ 150i, Thermo Scientific, Waltham, MA, USA). Subculturing was performed at 80% confluency using 0.25% trypsin-EDTA (Gibco, 25200072) with a 1:4 split ratio. Media were refreshed every 48 h.

#### 2.6.2. Oxygen–Glucose Deprivation (OGD)

The cells (passages 8–15) were seeded in 6-well plates (2 × 10^5^ cells/well) and grown to 90% confluency. Culture medium was replaced with glucose-free, serum-free DMEM (Gibco, 11966025) pre-equilibrated with 1% O_2_/5% CO_2_/94% N_2_ (MACS VA500 microaerophilic workstation, Don Whitley Scientific, Bingley, UK). The cells were exposed to hypoxia (1% O_2_) at 37 °C for 4 h. OGD medium was replaced with normoxic complete DMEM, and the cells were returned to 5% CO_2_/95% air for 24 h recovery [22].

### 2.7. Flow Cytometry Analysis of Apoptosis and Mitochondrial Membrane Potential

Apoptosis and mitochondrial membrane potential (ΔΨm) in the HT22 cells were assessed via flow cytometry 24 h post-reoxygenation. For apoptosis analysis, the cells were harvested using non-enzymatic dissociation buffer, stained with Annexin V-FITC and propidium iodide (PI) (BD Biosciences, Franklin Lakes, NJ, USA, 556547) in binding buffer (15 min, 25 °C in darkness), and analyzed using a BD FACSCelesta™ flow cytometer (488 nm excitation; FITC: 530/30 nm, PI: 610/20 nm). Viable (Annexin V^−^/PI^−^), early apoptotic (Annexin V^+^/PI^−^), and late apoptotic/necrotic (Annexin V^+^/PI^+^) populations were quantified from 10,000 events per sample using FlowJo v10.8. ΔΨm was evaluated via JC-1 staining (Cayman Chemical, Ann Arbor, MI, USA, 10009172); the cells incubated with 2 μM JC-1 (20 min, 37 °C) were analyzed for fluorescence shifts, with high ΔΨm values indicated by JC-1 aggregates (590 nm emission) and low ΔΨm values by monomers (530 nm emission). The ΔΨm index was calculated as the ratio of aggregate-to-monomer fluorescence intensity, validated by the CCCP (50 μM and 30 min)-treated positive controls.

### 2.8. Cell Staining

The cells grown on coverslips were fixed with 4% PFA (10 min, RT), permeabilized with 0.1% Triton X-100 (10 min), and blocked with 5% BSA (1 h). Primary antibodies against c-Fos (1:500) and MCT2 (1:200) (CST) were applied overnight at 4 °C. After PBS washing, the cells were incubated with fluorophore-conjugated secondary antibodies (1:1000, CST) for 1 h. Nuclei were counterstained with DAPI in ProLong Gold Antifade mountant (Thermo Fisher, Waltham, MA, USA). Images were acquired using an Olympus IX83 fluorescence microscope and Leica SP8 confocal system (Wetzlar, Germany). Fluorescence intensity, normalized to DAPI, was quantified via ImageJ using ≥5 random fields per sample.

### 2.9. Western Blot Analysis of Cellular Proteins

The HT22 cells were lysed in RIPA buffer supplemented with protease/phosphatase inhibitors. Proteins (20–30 µg) separated via 10% SDS-PAGE (70 V stacking, 120 V resolving; Bio-Rad, Hercules, CA, USA) were transferred to PVDF membranes (200 mA and 90 min). The membranes blocked with 5% non-fat milk/TBST were probed with primary antibodies against LDH1, HK, PFK1, PKM1, and GAPDH (1:1000–1:5000, CST) overnight at 4 °C, followed by HRP-conjugated secondary antibodies (1:5000, CST). Signals were visualized using ECL (Bio-Rad) on a ChemiDoc XRS+ system. Band intensities (ImageJ) were normalized to GAPDH for quantification.

### 2.10. Statistical Analysis

Statistical analyses were performed using GraphPad Prism 8.0 (GraphPad Software, San Diego, CA, USA), MATLAB (version R2022a, with a custom-developed NMRSpec module for spectral quantification), and SPSS 21.0 (IBM, Armonk, NY, USA). The normality of data distribution was confirmed via the Kolmogorov–Smirnov test (α = 0.05) prior to parametric analyses. Group differences were assessed by one-way analysis of variance (ANOVA), with Tukey’s post hoc test applied for pairwise comparisons, where ANOVA yielded statistical significance (*p* < 0.05). For nonparametric or nonlinear associations, Spearman’s rank correlation coefficient (ρ) was calculated to evaluate the monotonic relationships between variables. All the quantified values are expressed as mean ± standard error of the mean (SEM). The visualization of the statistical results, including bar graphs, correlation plots, and density distributions, was implemented using the ggplot2 package in R (version 4.2.3).

## 3. Results

### 3.1. Lactate Serves as a Compensatory Metabolic Hub with Inter-Regional Coordination in Ischemic Brains

**Figure 1:**
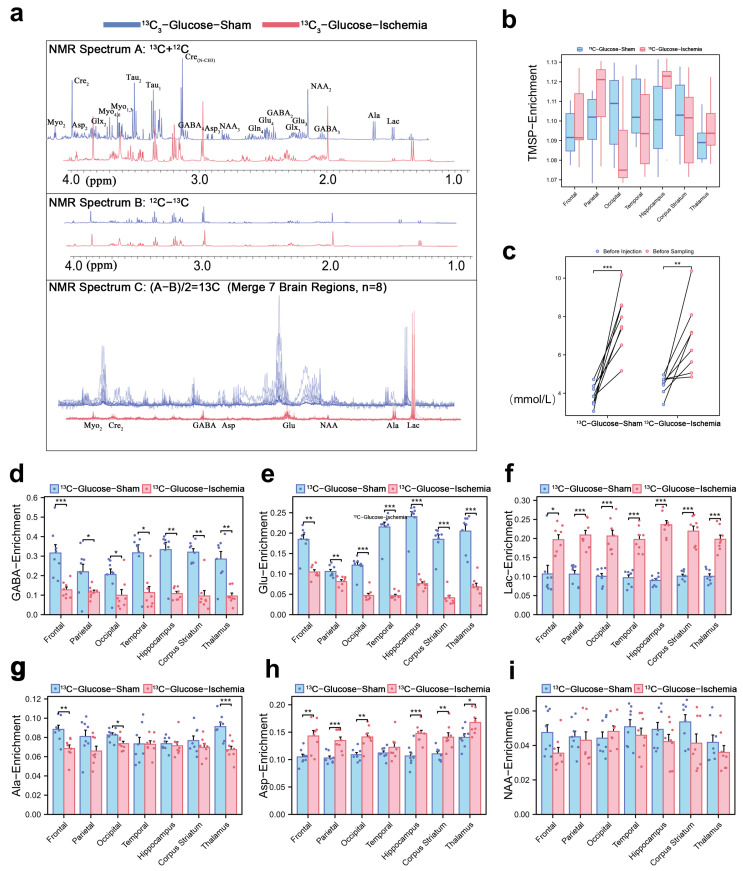
Cerebral metabolic rewiring in ischemic mice via ^1^H-^13^C NMR and isotopic enrichment profiling (**a**) Representative ^1^H-^13^C NMR spectrum (600 MHz, 25 °C) of cortical extracts (1–4 ppm region). Cₙ denotes ^13^C labeling at *n*-th carbon (e.g., Lac-C3: enrichment at lactate’s third carbon). (**b**) Normalized ^31^P-TMSP (trimethylsilylpropionate) enrichment ratios during isotopic steady-state, validating tracer equilibration. (**c**) Blood glucose levels pre-injection and pre-sacrifice. (**d**,**e**) ^13^C-enrichment ratios of key metabolites across experimental groups in different brain regions, expressed as [^13^C]/ [^13^C + ^12^C] ×100%. Regional ^13^C-enrichment of (**d**) GABA, (**e**) glutamate, (**f**) lactate, (**g**) Alanine, (**h**) Aspartate, and (**i**) N-acetylaspartate in different brain regions. (* *p* < 0.05, ** *p* < 0.01, *** *p* < 0.001).

The ^1^H-^13^C NMR spectrum (1–4 ppm, Figure 1a) reflects the key cerebral metabolites based on their chemical shifts and structural characteristics. Normalized TMSP enrichment (1.10 ± 0.03, Figure 1b) confirmed isotopic equilibrium, while the pre-sacrifice blood glucose fluctuations (Figure 1c) validated the sustained metabolic labeling of infused substrates (glucose or lactate). The quantification of ^13^C-enrichment ratios via spectral peak integration (Figure 1d,e) revealed global reductions in the number of cerebral metabolites under ischemia. Concurrently, the key excitatory neurotransmitter GABA and inhibitory glutamate showed consistent depletion (~50% reduction across all the brain regions), indicating their diminished capacity to sustain post-ischemic neuronal signaling. However, the lactate enrichment level remained elevated (Figure 1f), demonstrating both its rapid intra-ischemic accumulation and its critical role as a compensatory metabolic substrate in ischemic brain tissue. Notably, we selected several lactate-related three-carbon units, Alanine, Aspartate, and N-acetylaspartate, and their enrichment levels remained consistent (Figure 1f,g,i).

**Figure 2:**
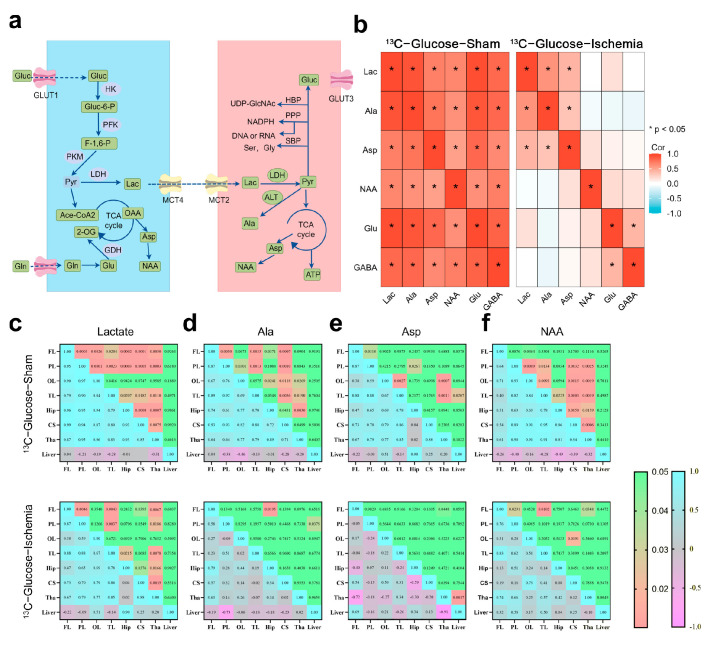
Metabolic pathway dynamics and regional coordination in ischemic brain (**a**) Schematic of astrocytic–neuronal metabolic coupling highlighting key enzymes and pathways (glutamate–glutamine cycle and lactate shuttle). (**b**) Correlation heatmap illustrating inter-metabolite relationships within same sample. Key representative metabolites were selected for analysis. Correlation coefficient (*R*) greater than 0.8 and *p*-value less than 0.05 were considered to indicate significant correlations. (**c**–**f**) Correlation analysis of specific metabolites across different brain regions. Upper panel shows *p*-values, while lower panel maps correlation coefficients (*R*), with significant correlations defined as *R* > 0.8 and *p* < 0.05. Heatmaps were constructed using ggplot for better visualization. All correlation data were obtained through tests that assume normal distribution.

Based on the NMR metabolic profiles, we mapped the pathways involving the substrates with sustained enrichment under ischemia using a schematic network (Figure 2a). To assess metabolic network stability, Spearman correlation analyses (|*R*| > 0.8, FDR-adjusted *p* < 0.01) were conducted on the paired metabolites within identical samples (Figure 2b). The pre-ischemic brains exhibited extensive connectivity across the central carbon metabolism pathways (e.g., TCA cycle intermediates and amino acid biosynthesis). Post-ischemia, this coordination was disrupted, with only lactate, Aspartate (Asp), and Alanine (Ala) maintaining significant correlations (R > 0.80). The retained Lactate–Ala correlation implies persistent substrate-level phosphorylation, while Asp connectivity suggests residual nitrogen shuttle activity.

The brain regions showed coherent metabolite dynamics under physiological conditions (Figure 2c–f), whereas ischemia abolished this synchrony. Despite global network fragmentation, lactate retained the inter-regional exchange capacity (cortex–striatum–thalamus). The thalamus demonstrated stronger metabolic connectivity compared to that of the cortical regions positioning it as a potential metabolic hub during ischemic stress.

### 3.2. Lactate Attenuates Behavioral Deficits and Stabilizes Mitochondrial Integrity in Ischemic Mice

**Figure 3:**
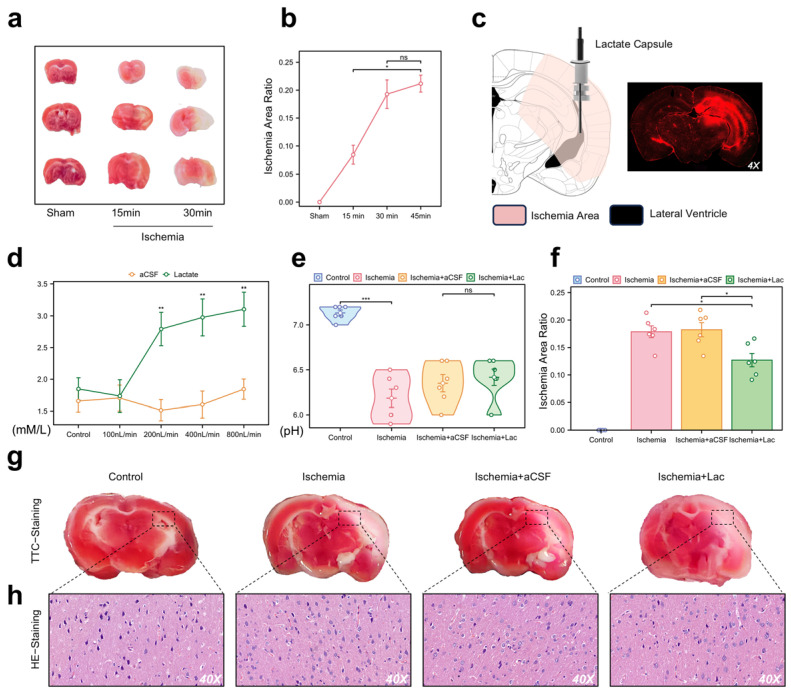
Pathological validation and intraventricular lactate intervention in acute cerebral ischemia (**a**) Representative 2,3,5-triphenyltetrazolium chloride (TTC) staining of coronal brain sections (*n* = 6/group). White regions indicate ischemic infarction. (**b**) Temporal quantification of infarct volume (percentage of hemisphere) using ImageJ (v1.53). Data are expressed as mean ± SEM. (**c**) Stereotaxic schematic of dual-lumen cannula implantation in lateral ventricle (LV). Fluorescence imaging (right) confirms diffusion range of Texas Red dextran infused via cannula. (**d**) CSF lactate concentrations monitored through cannula pressure ports during continuous LV infusion (0–10 mM gradient). (**e**) Cerebral pH measurement. No significant intergroup differences detected (*p* > 0.1). (**f**) Quantification of infarct area in each group. Infarct area was measured and expressed as percentage of total brain area. (**g**) TTC staining images of each group. (**h**) Hematoxylin and eosin (H&E) staining demonstrating neuronal pathology (40× magnification). (ns: * *p* > 0.05, * *p* < 0.05, ** *p* < 0.01, *** *p* < 0.001).

To validate our in vivo model for the NMR analysis of ischemic brain regions, we used an acute hemispheric ischemia model of mice, which induces infarction in most brain areas within 30 min (Figure 3a,b). Three weeks before the experiment, we implanted a dual-chamber catheter into each mouse’s skull, secured with a cranial screw and dental cement. The photosensitive dye injected into the lateral ventricle spread to the adjacent brain regions, corresponding to the TTC staining distribution (Figure 3c). We optimized the infusion rate for lateral ventricle administration at 400 nL/min, which was maintained during ischemia, achieving saturation without altering the pH levels (Figure 3d,e). The infarct area in the lactate group was smaller than that in the other groups (Figure 3f,g). Pathological analysis showed significant neuronal damage in the ischemic group, with lactate-supplemented CSF demonstrating a potential therapeutic effect (Figure 3h).

**Figure 4:**
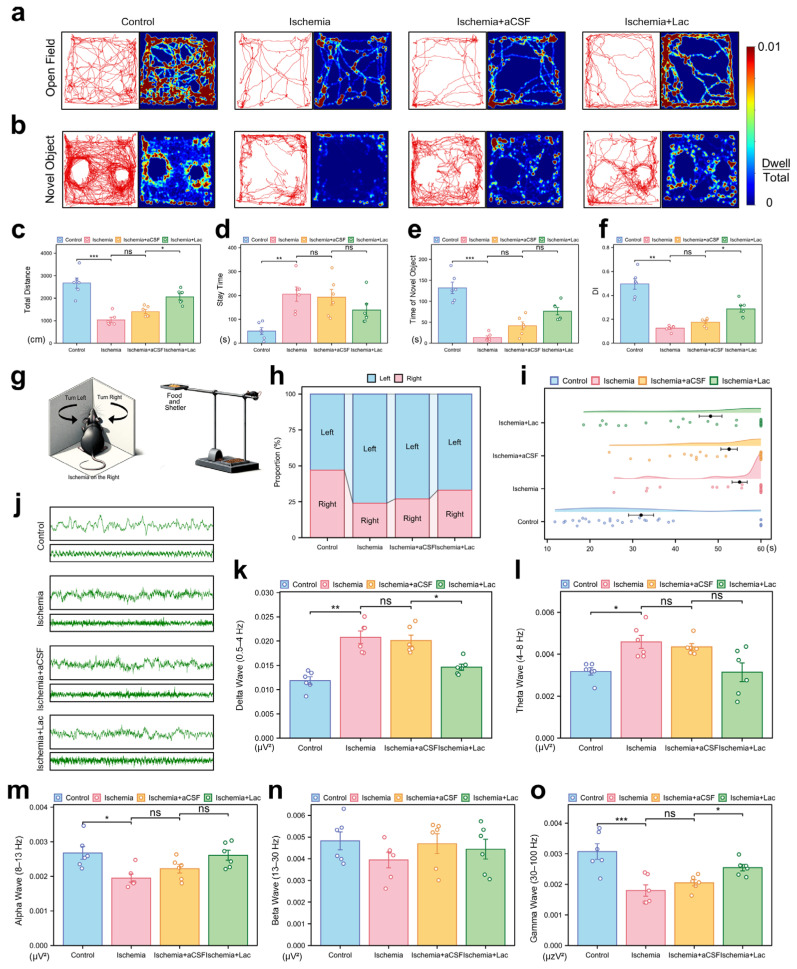
Behavioral and neurophysiological consequences of cerebral ischemia (**a**) Open field trajectory heatmaps. Color intensity reflects spatial dwell time. (**b**) Novel object recognition (NOR) exploration patterns. Red circles: novel object; blue circles: familiar object. (**c**,**d**) Open field quantification: (**c**) total locomotion distance (cm); (**d**) center zone dwell time (%). (**e**,**f**) NOR analysis: (**e**) novel object interaction time; (**f**) discrimination index (DI = [Novel − Familiar]/Total). (**g**) Schematic representation of corner test and elevated maze test. (**h**) Turn ratio in corner test. (**i**) Time spent on elevated single-bridge task for each group of mice, shown as raincloud plot. Mice were required to cross elevated single bridge, and time taken to complete task was recorded. Mice that fell off or did not complete task within 60 s were assigned time of 60 s. (**j**) Representative electrocorticography traces: 2 s (upper) and 10 s (lower) epochs. (**k**–**o**) Spectral power analysis: (**k**) Delta (0.5–4 Hz), (**l**) Theta (4–8 Hz), (**m**) Alpha (8–13 Hz), (**n**) Beta (13–30 Hz), and (**o**) Gamma (30–100 Hz). Bar plots show integrated power (μV^2^/Hz). (ns: * *p* > 0.05, * *p* < 0.05, ** *p* < 0.01, *** *p* < 0.001).

Behavioral analyses demonstrated that cerebral ischemia significantly impaired motor coordination and exploratory activity, with the ischemic mice exhibiting reduced locomotion (*p* < 0.001) and prolonged immobility in the open field tests (*p* < 0.01). Intraventricular lactate supplementation partially restored their locomotor function, increasing the level of exploratory behavior (*p* < 0.05) (Figure 4a,c,d). Cognitive deficits were evident in the novel object recognition tasks, as ischemia lowered the discrimination index (DI, *p* < 0.01), indicating diminished memory retention. Lactate administration ameliorated this decline, improving the DI to the near-baseline levels (*p* < 0.05). All the behavioral outcomes were validated across three independent replicates (Figure 4b,e,f). Ischemia induced a significant imbalance in the turning behavior of the mice in the corner test, with a pronounced shift towards leftward turning after right cerebral ischemia. This suggests impaired motor function in the left limbs due to damage to the right hemisphere. Notably, the lactate-treated group exhibited a significant recovery in this turning behavior (Figure 4h). In the elevated single plank bridge test, ischemia led to a marked reduction in the success rate of the mice crossing the bridge, with many mice unable to reach the shelter containing food, reflecting severe deficits in motor coordination and balance. However, lactate infusion significantly ameliorated these deficits, as evidenced by their improved performance in the task (Figure 4i).

The electrophysiological recordings revealed severe post-ischemic neural dysregulation, characterized by aberrant high-amplitude delta oscillations (0.5–4 Hz, *p* < 0.01) and suppressed gamma activity (30–100 Hz, *p* < 0.001), consistent with pathological hyperactivity and disrupted cortical communication. Lactate intervention attenuated these abnormalities, notably restoring theta (4–8 Hz, *p* < 0.05), which correlates with behavioral recovery. These findings collectively suggest lactate mitigates ischemia-induced neural network destabilization through frequency-specific modulation (Figure 4j–o).

**Figure 5:**
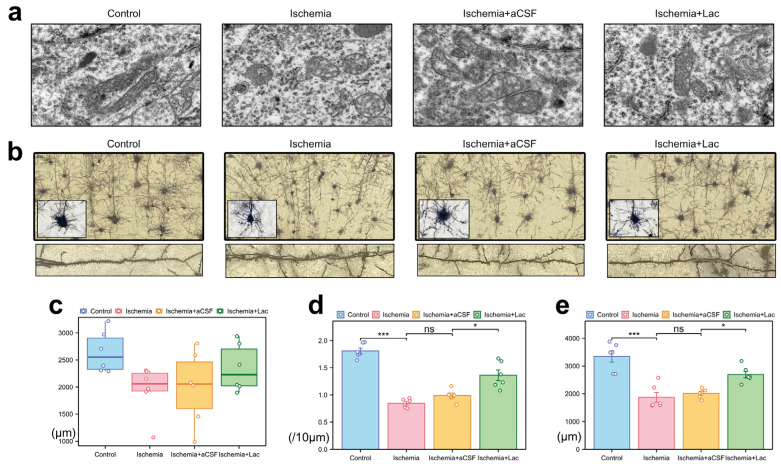
Ischemia-induced ultrastructural and neuronal morphological alterations (**a**) Mitochondrial pathology (TEM) (10,000×). (**b**) Neuronal morphology (Golgi–Cox staining) (40×, upper; 100×, lower). (**c**) Axonal degeneration: quantification of total axonal length via ImageJ skeletonization. (**d**) Dendritic spine loss: spine density (spines/10 μm) in secondary dendrites (50–150 μm from soma) measured with Simple Neurite Tracer (Fiji). (**e**) Dendritic simplification: Sholl analysis of total dendritic length (10 μm radial intervals) reveals ischemia-induced arborization deficits (ns: * *p* > 0.05, * *p* < 0.05, *** *p* < 0.001).

Following cerebral ischemia, neuronal mitochondria exhibited marked ultrastructural damage, including swelling, cristae fragmentation, and matrix disorganization (Figure 5a). The lateral ventricular infusion of lactate-enriched artificial cerebrospinal fluid (aCSF) partially reversed these pathologies, restoring mitochondrial elongation and cristae integrity. Ischemic injury induced severe dendritic atrophy, characterized by reduced neurite complexity and disorganized axodendritic trajectories (Figure 5b). While the total axonal length remained statistically unchanged, the focal varicosities and the cytoskeletal discontinuities suggested subcellular axonal degeneration (Figure 5c). Notably, lactate intervention ameliorated synaptic plasticity deficits, more significantly increasing dendritic spine density and total spine length compared to those of the aCSF controls (Figure 5d,e). The preferential recovery of dendritic spines—the dynamic hubs of synaptic activity—highlights their heightened metabolic vulnerability to ischemic stress and responsiveness to lactate-mediated bioenergetic support, likely through the stabilization of mitochondrial function and ATP-dependent cytoskeletal remodeling.

### 3.3. pH-Neutral Lactate Confers Ischemic Tolerance via Glycolytic Reprogramming and H^+^ Homeostasis

**Figure 6:**
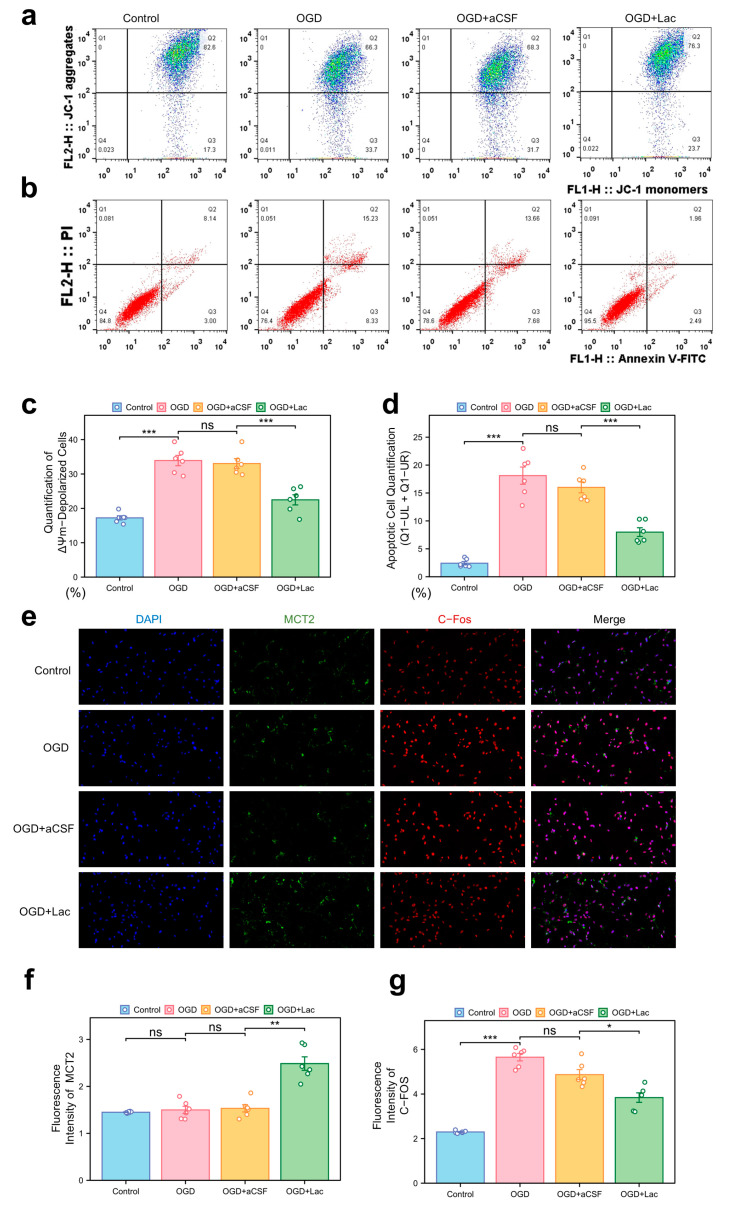
Mitochondrial dysfunction, apoptosis, and neuronal activity under ischemia with lactate intervention (**a**) Mitochondrial membrane potential (ΔΨm) by JC-1 staining (flow cytometry). X-axis: JC-1 monomers (FL1-H); Y-axis: JC-1 aggregates (FL2-H). Q1: ΔΨm-depolarized cells (monomers↑/aggregates↓); Q2: ΔΨm-hyperpolarized cells (monomers↓/aggregates↑). (**b**) Apoptosis analysis via Annexin V-FITC/PI dual staining. X-axis: Annexin V-FITC (FL1-H, apoptosis marker); Y-axis: PI (FL3-H, necrosis marker). Q1-UR: Late apoptotic (Annexin V+/PI+); Q1-LR: Necrotic (Annexin V−/PI+); Q1-UL: Early apoptosis (Annexin V+/PI−). Lactate reduces Q1-UL/Q1-UR populations. (**c**) Quantification of ΔΨm-depolarized cells (Q1 percentage). (**d**) Apoptotic cell quantification (Q1-UL + Q1-UR). (**e**) Immunofluorescence of c-Fos (neuronal activity) and MCT2 (lactate transporter). (20×). (**f**,**g**) Quantitative analysis normalized to DAPI: (**f**) MCT2 mean fluorescence intensity (MFI), (**g**) c-Fos mean fluorescence intensity. (ns: * *p* > 0.05, * *p* < 0.05, ** *p* < 0.01, *** *p* < 0.001).

In the HT22 hippocampal neuronal models subjected to oxygen–glucose deprivation (OGD), mitochondrial dysfunction was evident through JC-1 staining, with a pronounced shift toward ΔΨm depolarization (Figure 6a). Lactate supplementation (OGD + LAc) significantly attenuated this loss, restoring mitochondrial polarization more compared to that of the OGD and OGD + aCSF controls. Apoptosis analysis revealed elevated Annexin V+ populations in the OGD group, while lactate intervention reduced both the early and late apoptotic cell fractions (Figure 6b,d). OGD-induced neuronal hyperexcitation was evidenced by c-Fos upregulation, a marker of excitotoxic stress, which was significantly attenuated in the lactate-treated group (*p* < 0.05) (Figure 6e,g). In contrast, MCT2 expression—a rate-limiting transporter for neuronal lactate uptake—showed delayed ischemia-induced upregulation, while lactate intervention accelerated and amplified this response (*p*< 0.05) (Figure 6e,f).

**Figure 7:**
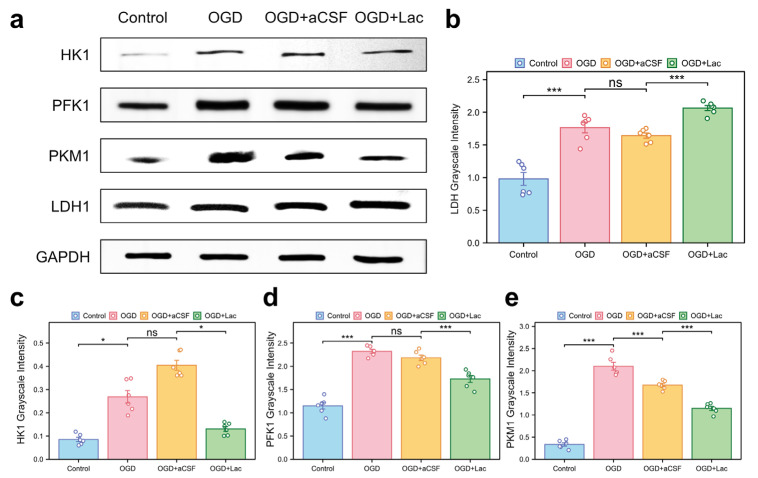
Glycolytic enzyme expression profiles in OGD-treated HT22 (**a**) Western blot analysis of key glycolytic enzymes. (**b**–**e**) Quantitative densitometry normalized to GAPDH: (**b**) lactate dehydrogenase (LDH); (**c**) hexokinase 1 (HK1); (**d**) phosphofructokinase 1 (PFK1); and (**e**) pyruvate kinase M1 (PKM1) (ns: * *p* > 0.05, * *p* < 0.05, *** *p* < 0.001).

Western blot analysis revealed significant glycolytic remodeling in the OGD-treated HT22 hippocampal neurons. Oxygen–glucose deprivation (OGD) upregulated the expressions of lactate dehydrogenase (LDH), phosphofructokinase 1 (PFK1), pyruvate kinase M1 (PKM1), and hexokinase 1 (HK1) more compared to those of the controls (*p* < 0.05). Lactate intervention further elevated the LDH levels († *p* < 0.001 vs. OGD), while paradoxically suppressing PFK1, PKM1, and HK1 expression († *p* < 0.05) (Figure 7).

## 4. Discussion

One of the critical challenges in brain ischemia treatment lies in the need for more comprehensive therapeutic approaches that can simultaneously address the various facets of ischemic damage [8]. The current treatments often focus on isolated mechanisms, failing to comprehensively mitigate the full spectrum of neuronal injury and metabolic dysfunction induced by ischemia [23]. As neuronal damage becomes largely irreversible once it progresses beyond a certain point, there is an urgent need for novel therapies capable of alleviating this damage and promoting recovery [24].

The brain is highly sensitive to ischemia, with the complete interruption of blood flow for 5–10 s leading to the loss of perception or consciousness [25]. The metabolic network collapse and compensatory mechanisms triggered by brain ischemia are the core elements of the stroke pathological process. With the continuous development of our understanding of brain functional networks, our recognition of ischemia should be elevated to encompass the metabolic processes across the entire brain.

In this study, we employed ^1^H-^13^C nuclear magnetic resonance (NMR) to investigate the enrichment and correlation of various metabolites in different brain regions following hemispheric ischemia. By integrating electrophysiological recordings with ultrastructural analysis, we systematically revealed the multi-layered characteristics of neuronal metabolic reprogramming under acute ischemic conditions. Unlike the previous studies focusing on the changes in individual metabolites, we observed that ischemia-induced hypoxia and glucose deprivation led to a decrease in the enrichment of key metabolites. However, several smaller three-carbon units (such as Lactic acid (Lac), Alanine (Ala), Aspartate (Asp), and N-acetylaspartate (NAA)) showed either higher or unchanged enrichment levels (Figure 2). We refer to these metabolites as the “minimum essential units”, whose formation is essential as primary metabolic products during cellular emergency responses.

Lactate plays a dual role in this metabolic reprogramming; it is both a byproduct of terminal glycolysis and a key mediator in maintaining inter-regional energy balance [10]. NMR correlation network analysis revealed that following ischemia, only lactate retains the ability to be transferred across brain regions, with the thalamus serving as the core hub of metabolic compensation (Figure 2c). The high metabolic activity in the thalamus may be related to its inherent astrocyte–neuron lactate shuttle (ANLS) system density, and decreased metabolic coupling leading to thalamocortical disconnection may be a critical factor contributing to post-stroke consciousness impairment [26]. In line with this, the behavioral experiments showed that lactate intervention significantly restored brain functional connectivity (Figure 4c) and improved both the motor and cognitive functions (Figure 4c–f). This finding provides a metabolic basis for the therapeutic effects of the deep brain stimulation (DBS) of the thalamus in clinical stroke rehabilitation [27].

At the cellular level, lactate plays a synergistic role in both energy rescue and signal regulation. On one hand, lactate is taken up via MCT2-dependent transportation (Figure 6e,f), replenishing carbon sources for the TCA cycle and helping to maintain the mitochondrial membrane potential (Figure 6a). Notably, lactate also exhibits the bidirectional regulation of glycolytic flux (Figure 7). The upregulation of lactate dehydrogenase (LDH) expression (Figure 7b) accelerates the conversion of pyruvate to lactate, thereby facilitating glycolysis under hypoxic conditions. Conversely, the downregulation of phosphofructokinase-1 (PFK1) (Figure 7d) may help alleviate osmotic stress by reducing the accumulation of fructose-1,6-bisphosphate (F-1,6-BP), a potent osmolyte. This reduction in F-1,6-BP accumulation could also lead to a decrease in proton (H^+^) production, further helping to alleviate acidotic stress. This finely tuned “end-product acceleration and upstream inhibition” mechanism not only provides insight into the metabolic adaptations to ischemic stress, but also opens new avenues for therapeutic interventions aimed at regulating the metabolic rhythms in pathological conditions, such as stroke or neurodegenerative diseases [28].

Under cerebral ischemia, the neurons upregulate hexokinase (HK), phosphofructokinase 1 (PFK1), pyruvate kinase M1 (PKM1), and lactate dehydrogenase (LDH) through HIF-1α/mTORC1 signaling, driving global glycolytic flux amplification to rapidly generate ATP and regenerate NAD+ for sustaining anaerobic glycolysis [29]. However, this high-flux, low-efficiency metabolic pattern exacerbates neuronal damage by inducing lactate overaccumulation and intracellular acidosis [30]. Lateral ventricular lactate supplementation remodels metabolic homeostasis via dual mechanisms: (i) Lactate suppresses PI3K/Akt/mTOR signaling to destabilize HIF-1αand allosterically inhibits PFK1 activity, thereby reducing HK, PFK1, and PKM1 expression to alleviate osmotic stress from glycolytic overload [31]; (ii) lactate drives LDH reverse catalysis (lactate→pyruvate) through mass action effects, while upregulating LDHB isoform expression, enhancing pyruvate regeneration capacity to sustain NAD+ recycling and mitochondrial oxidation. This bidirectional reprogramming not only mitigates glycolysis-dependent acidosis and oxidative stress, but also partially restores energy homeostasis by reviving pyruvate-TCA cycle flux, ultimately blocking mitochondrial permeability transition pore opening and Caspase-3-dependent apoptosis [30]. These findings provide a mechanistic foundation for optimizing neuroprotection strategies through targeting metabolic nodes.

The dual role of lactate in cerebral ischemia presents both significant challenges and opportunities for therapeutic intervention [32]. Clinically, lactate accumulation is commonly regarded as a marker of metabolic crisis, with primary attention given to the correction of acidosis [33]. However, such an approach risks neglecting lactate’s more complex role as an alternative metabolic substrate during energy deprivation [34]. Our findings suggest that lactate does not solely function as a fuel source, but also exerts neuroprotective effects through the activation of the key signaling pathways, particularly via the HCAR1 receptor and the downstream PI3K/AKT and MAPK cascades [16,35,36]. These pathways converge to promote mitochondrial biogenesis and suppress apoptosis, key processes for maintaining neuronal survival under ischemic conditions. Moreover, lactate inhibits OGD-induced intracellular calcium overload in neurons and astrocytes, reduces cell death, and shifts metabolism toward aerobic oxidation, while also mitigating pro-inflammatory responses in ischemic and inflammatory environments [37].

One of the most intriguing aspects of lactate’s function is its ability to activate AMP-activated protein kinase (AMPK) [38], which stabilizes cellular energy homeostasis. This activation creates a feedforward loop that supports glycolytic–astrocytic coupling even under ischemic conditions. These mechanisms highlight the potential for lactate supplementation to address the root cause of metabolic collapse, moving beyond mere acidosis mitigation. Our study provides compelling evidence that lactate can be leveraged as a therapeutic agent, not just as a byproduct of anaerobic metabolism, but as a crucial mediator of cellular resilience during ischemia.

The clinical relevance of these findings lies in the method of lactate delivery. To isolate the effects of lactate anions from proton-mediated acidotoxicity, we administered pH-neutral sodium lactate (7.4), a distinction not typically made in the prior investigations. This approach mirrors emerging clinical strategies that use balanced electrolyte solutions containing L-lactate (e.g., Hartmann’s solution) [39], which maintain the physiological pH, while delivering bioavailable lactate. Importantly, the absence of systemic pH perturbation in our model suggests that the benefits observed are due to lactate anions themselves, rather than the buffering effects of pH correction.

These findings challenge the conventional view of lactate as either a toxic byproduct or a passive energy substrate. Instead, we propose that lactate functions as a metabolic orchestrator, capable of reprogramming ischemic neurochemistry through both the bioenergetic and signaling pathways. This paradigm shift opens new possibilities for treating ischemic neurodegeneration. To further optimize lactate-based therapies, future research should explore targeted delivery strategies, such as intra-arterial infusion to facilitate direct cerebral uptake, or the use of nanoparticle-encapsulated lactate to overcome the blood–brain barrier [40]. Additionally, combining lactate with the inhibitors of monocarboxylate transporter (MCT) saturation, such as quercetin derivatives, may enhance its therapeutic efficacy by increasing lactate availability in ischemic tissues [41].

In summary, our study underscores the importance of reevaluating lactate’s role in cerebral ischemia. By redefining lactate from a mere waste product to precision metabolic therapy, we offer new insights into the potential for lactate to mitigate ischemic neurodegeneration and provide a foundation for future clinical applications. Further investigations into optimal delivery routes and combination strategies will be essential for translating these findings into effective therapies for ischemic brain injury.

## 5. Conclusions

This study combines in vivo and cellular analyses with ^13^C-labeled liquid NMR to investigate metabolic changes in brain tissue following hemispheric ischemia. It identifies lactate not only as a critical metabolic substrate in ischemic brain injury, but also as a potential protector against such damage. In summary, this study underscores the complex dual role of lactate in cerebral ischemia. While lactate accumulation has traditionally been associated with acidosis, it also serves as a crucial metabolic substrate for energy production. By administering sodium lactate in a pH-controlled environment, this study effectively isolates the beneficial metabolic effects of lactate anions from the potential harm caused by acidosis. These findings highlight lactate’s therapeutic potential in enhancing bioenergetic recovery and offer new insights into its clinical application in ischemic diseases.

## Figures and Tables

**Table 1:** Chemical shift ranges for different brain metabolites.

Metabolism	Center	From	To
TMSP	0.004	0.106	−0.097
Lactate	1.328	1.354	1.301
GABA3	1.965	1.980	1.950
GABA4	2.299	2.274	2.324
NAA	2.019	2.031	2.006
NAAG	2.053	2.060	2.045
Glu3	2.070	2.077	2.063
Glu4	2.355	2.385	2.325
Gln4	2.438	2.460	2.416
Glx2	3.770	3.810	3.730
Glx3	2.147	2.200	2.094
Suc	2.404	2.411	2.397
Asp3	2.742	2.853	2.631
m-Ins	4.063	4.082	4.044
m-Ins2	3.273	3.298	3.248
m-Ins3	3.584	3.510	3.659
tau	3.427	3.457	3.397
Ala	1.487	1.510	1.465

## Data Availability

The raw data supporting the conclusions of this article will be made available by the authors on request.

## References

[1] Rabinowitz J.D., Enerback S. (2020). Lactate: The ugly duckling of energy metabolism. Nat. Metab..

[2] De Tymowski C., Soussi S., Depret F., Legrand M. (2017). On-line plasma lactate concentration monitoring in critically ill patients. Crit. Care.

[3] Crapnell R.D., Tridente A., Banks C.E., Dempsey-Hibbert N.C. (2021). Evaluating the Possibility of Translating Technological Advances in Non-Invasive Continuous Lactate Monitoring into Critical Care. Sensors.

[4] Hilkens N.A., Casolla B., Leung T.W., de Leeuw F.E. (2024). Stroke. Lancet.

[5] Zhao Y., Zhang X., Chen X., Wei Y. (2022). Neuronal injuries in cerebral infarction and ischemic stroke: From mechanisms to treatment (Review). Int. J. Mol. Med..

[6] Otto C., Kalantzis R., Kubler-Weller D., Kuhn A.A., Bold T., Regler A., Strathmeyer S., Wittmann J., Ruprecht K., Heelemann S. (2024). Comprehensive analysis of the cerebrospinal fluid and serum metabolome in neurological diseases. J. Neuroinflammation.

[7] Iadecola C., Smith E.E., Anrather J., Gu C., Mishra A., Misra S., Perez-Pinzon M.A., Shih A.Y., Sorond F.A., van Veluw S.J. (2023). The Neurovasculome: Key Roles in Brain Health and Cognitive Impairment: A Scientific Statement From the American Heart Association/American Stroke Association. Stroke.

[8] Rundek T., Chen C.L.H. (2023). Advances in Stroke: Brain Health in 2023. Stroke.

[9] Baranovicova E., Kalenska D., Kaplan P., Kovalska M., Tatarkova Z., Lehotsky J. (2023). Blood and Brain Metabolites after Cerebral Ischemia. Int. J. Mol. Sci..

[10] Ye L., Jiang Y., Zhang M. (2022). Crosstalk between glucose metabolism, lactate production and immune response modulation. Cytokine Growth Factor. Rev..

[11] Bhatti M.S., Frostig R.D. (2023). Astrocyte-neuron lactate shuttle plays a pivotal role in sensory-based neuroprotection in a rat model of permanent middle cerebral artery occlusion. Sci. Rep..

[12] Bouzat P., Oddo M. (2014). Lactate and the injured brain: Friend or foe?. Curr. Opin. Crit. Care.

[13] Dienel G.A. (2014). Lactate shuttling and lactate use as fuel after traumatic brain injury: Metabolic considerations. J. Cereb. Blood Flow Metab..

[14] Qiu L.L., Tan X.X., Yang J.J., Ji M.H., Zhang H., Zhao C., Xia J.Y., Sun J. (2023). Lactate Improves Long-term Cognitive Impairment Induced By Repeated Neonatal Sevoflurane Exposures Through SIRT1-mediated Regulation of Adult Hippocampal Neurogenesis and Synaptic Plasticity in Male Mice. Mol. Neurobiol..

[15] Wei J.P., Wen W., Dai Y., Qin L.X., Wen Y.Q., Duan D.D., Xu S.J. (2021). Drinking water temperature affects cognitive function and progression of Alzheimer’s disease in a mouse model. Acta Pharmacol. Sin..

[16] Huang Y.F., Wang G., Ding L., Bai Z.R., Leng Y., Tian J.W., Zhang J.Z., Li Y.Q., Qin Y.H., Ahmad (2023). Lactate-upregulated NADPH-dependent NOX4 expression via HCAR1/PI3K pathway contributes to ROS-induced osteoarthritis chondrocyte damage. Redox Biol..

[17] Ojo O.B., Amoo Z.A., Olaleye M.T., Jha S.K., Akinmoladun A.C. (2023). Time and Brain Region-Dependent Excitatory Neurochemical Alterations in Bilateral Common Carotid Artery Occlusion Global Ischemia Model. Neurochem. Res..

[18] Kawalec M., Wojtyniak P., Bielska E., Lewczuk A., Boratynska-Jasinska A., Beresewicz-Haller M., Frontczak-Baniewicz M., Gewartowska M., Zablocka B. (2023). Mitochondrial dynamics, elimination and biogenesis during post-ischemic recovery in ischemia-resistant and ischemia-vulnerable gerbil hippocampal regions. Biochim. Biophys. Acta Mol. Basis Dis..

[19] Bagga P., Behar K.L., Mason G.F., De Feyter H.M., Rothman D.L., Patel A.B. (2014). Characterization of cerebral glutamine uptake from blood in the mouse brain: Implications for metabolic modeling of 13C NMR data. J. Cereb. Blood Flow Metab..

[20] Govind V., Young K., Maudsley A.A. (2000). Corrigendum: Proton NMR chemical shifts and coupling constants for brain metabolites. NMR Biomed..

[21] Du F. (2019). Golgi-Cox Staining of Neuronal Dendrites and Dendritic Spines with FD Rapid GolgiStain Kit. Curr. Protoc. Neurosci..

[22] Zhao Y., Kong G.Y., Pei W.M., Zhou B., Zhang Q.Q., Pan B.B. (2019). Dexmedetomidine alleviates hepatic injury via the inhibition of oxidative stress and activation of the Nrf2/HO-1 signaling pathway. Eur. Cytokine Netw..

[23] DeSai C., Hays Shapshak A. (2023). Cerebral Ischemia. StatPearls.

[24] Wegener S., Baron J.C., Derdeyn C.P., Fierstra J., Fromm A., Klijn C.J.M., van Niftrik C.H.B., Schaafsma J.D. (2024). Hemodynamic Stroke: Emerging Concepts, Risk Estimation, and Treatment. Stroke.

[25] Madai S., Kilic P., Schmidt R.M., Bas-Orth C., Korff T., Buttner M., Klinke G., Poschet G., Marti H.H., Kunze R. (2024). Activation of the hypoxia-inducible factor pathway protects against acute ischemic stroke by reprogramming central carbon metabolism. Theranostics.

[26] Yamagata K. (2022). Lactate Supply from Astrocytes to Neurons and its Role in Ischemic Stroke-induced Neurodegeneration. Neuroscience.

[27] Tobimatsu S. (2022). Editorial: Neural oscillations in physiology and neuropsychiatric disorders. Front. Hum. Neurosci..

[28] Shen J., Chen A., Cai Z., Chen Z., Cao R., Liu Z., Li Y., Hao J. (2022). Exhausted local lactate accumulation via injectable nanozyme-functionalized hydrogel microsphere for inflammation relief and tissue regeneration. Bioact. Mater..

[29] Medel V., Crossley N., Gajardo I., Muller E., Barros L.F., Shine J.M., Sierralta J. (2022). Whole-brain neuronal MCT2 lactate transporter expression links metabolism to human brain structure and function. Proc. Natl. Acad. Sci. USA.

[30] Dong S., Qian L., Cheng Z., Chen C., Wang K., Hu S., Zhang X., Wu T. (2021). Lactate and Myocardiac Energy Metabolism. Front. Physiol..

[31] Li R., Zheng Y., Zhang J., Zhou Y., Fan X. (2023). Gomisin N attenuated cerebral ischemia-reperfusion injury through inhibition of autophagy by activating the PI3K/AKT/mTOR pathway. Phytomedicine.

[32] Collaborators G.B.D.S. (2021). Global, regional, and national burden of stroke and its risk factors, 1990-2019: A systematic analysis for the Global Burden of Disease Study 2019. Lancet Neurol..

[33] Braga A., Chiacchiaretta M., Pellerin L., Kong D., Haydon P.G. (2024). Astrocytic metabolic control of orexinergic activity in the lateral hypothalamus regulates sleep and wake architecture. Nat. Commun..

[34] Schurr A., Gozal E. (2011). Aerobic production and utilization of lactate satisfy increased energy demands upon neuronal activation in hippocampal slices and provide neuroprotection against oxidative stress. Front. Pharmacol..

[35] Laroche S., Stil A., Germain P., Cherif H., Chemtob S., Bouchard J.F. (2021). Participation of L-Lactate and Its Receptor HCAR1/GPR81 in Neurovisual Development. Cells.

[36] Li Z.C., Jia Y.P., Wang Y., Qi J.L., Han X.P. (2018). Effects of dexmedetomidine post-treatment on BDNF and VEGF expression following cerebral ischemia/reperfusion injury in rats. Mol. Med. Rep..

[37] Babenko V.A., Varlamova E.G., Saidova A.A., Turovsky E.A., Plotnikov E.Y. (2024). Lactate protects neurons and astrocytes against ischemic injury by modulating Ca(2+) homeostasis and inflammatory response. FEBS J..

[38] Liu P., Yang X., Hei C., Meli Y., Niu J., Sun T., Li P.A. (2016). Rapamycin Reduced Ischemic Brain Damage in Diabetic Animals Is Associated with Suppressions of mTOR and ERK1/2 Signaling. Int. J. Biol. Sci..

[39] Benke K., Jász D.K., Szilágyi Á.L., Baráth B., Tuboly E., Márton A.R., Varga P., Mohácsi Á., Szabó A., Széll Z. (2021). Methane supplementation improves graft function in experimental heart transplantation. J. Heart Lung Transplant. Off. Publ. Int. Soc. Heart Transplant..

[40] Shi Y., Zhao J., Li H., Yu M., Zhang W., Qin D., Qiu K., Chen X., Kong M. (2022). A Drug-Free, Hair Follicle Cycling Regulatable, Separable, Antibacterial Microneedle Patch for Hair Regeneration Therapy. Adv. Healthc. Mater..

[41] Zhao P., Wang S., Jiang J., Gao Y., Wang Y., Zhao Y., Zhang J., Zhang M., Huang Y. (2023). Targeting lactate metabolism and immune interaction in breast tumor via protease-triggered delivery. J. Control Release Off. J. Control Release Soc..

